# Competition of SARS-CoV-2 Variants in Cell Culture and Tissue: Wins the Fastest Viral Autowave

**DOI:** 10.3390/vaccines10070995

**Published:** 2022-06-22

**Authors:** Alexey Tokarev, Anastasia Mozokhina, Vitaly Volpert

**Affiliations:** 1 S.M. Nikolskii Mathematical Institute, Peoples Friendship University of Russia (RUDN University), 6 Miklukho-Maklaya Street, 117198 Moscow, Russia; mozokhina-as@rudn.ru (A.M.); volpert@math.univ-lyon1.fr (V.V.); 2 N.N. Semenov Federal Research Center for Chemical Physics RAS, 4 Kosygina Street, Building 1, 119991 Moscow, Russia; 3 Institut Camille Jordan, UMR 5208 CNRS, University Lyon 1, 69622 Villeurbanne, France

**Keywords:** viral infection, competition of viral strains, reaction–diffusion systems, autowaves, mathematical modeling and analysis

## Abstract

Replication of viruses in living tissues and cell cultures is a “number game” involving complex biological processes (cell infection, virus replication inside infected cell, cell death, viral degradation) as well as transport processes limiting virus spatial propagation. In epithelial tissues and immovable cell cultures, viral particles are basically transported via Brownian diffusion. Highly non-linear kinetics of viral replication combined with diffusion limitation lead to spatial propagation of infection as a moving front switching from zero to high local viral concentration, the behavior typical of spatially distributed excitable media. We propose a mathematical model of viral infection propagation in cell cultures and tissues under the diffusion limitation. The model is based on the reaction–diffusion equations describing the concentration of uninfected cells, exposed cells (infected but still not shedding the virus), virus-shedding cells, and free virus. We obtain the expressions for the viral replication number, which determines the condition for spatial infection progression, and for the final concentration of uninfected cells. We determine analytically the speed of spatial infection propagation and validate it numerically. We calibrate the model to recent experimental data on SARS-CoV-2 Delta and Omicron variant replication in human nasal epithelial cells. In the case of competition of two virus variants in the same cell culture, the variant with larger individual spreading speed wins the competition and eliminates another one. These results give new insights concerning the emergence of new variants and their spread in the population.

## 1. Introduction

**Figure 1:**
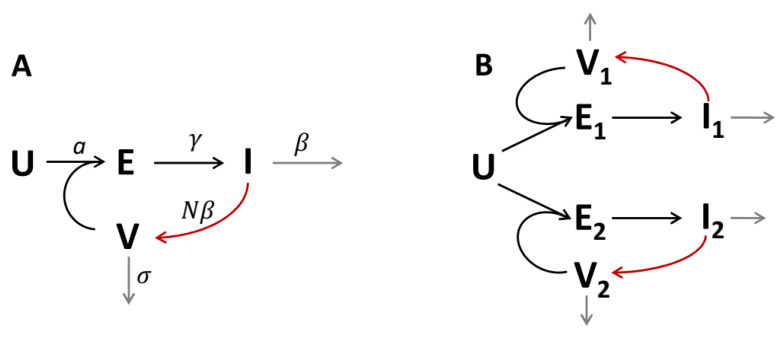
Schemes depicting the transitions of cells between different infected states (black arrows), virus production (red arrows), and death of cells and virus (gray arrows). **U**—uninfected cells; **E**—“exposed” cells (infected but still not shedding the virus); **I**—infected cells shedding the virus; **V**—free virus particles. (**A**) One-strain model. (**B**) Two-strain model with competition of strains for the uninfected cells.

Viruses are non-cellular pathogens which use cell machinery to self-replicate, resulting in infection having to do with the population, the organism, the local tissue, or—in the simplest case—just the cell culture ex vivo or in vitro. In any case, replication of the virus is a “number game” between complex but still local biological processes (cell infection, virus replication in an infected cell, virus release, cell death, viral degradation—see Figure 1A) and spatial processes of viral transport between cells (convection, diffusion). Without transport, infection cannot proceed. In this study, we are interested in pure diffusion control of viral replication, which can be expected if convection (in particular, blood and lymph flow) is negligible, which is the case during viral replication in epithelium in vivo and cell cultures ex vivo/in vitro. COVID-19 pandemics made this question actual, as SARS-CoV-2 virus replicates in the upper respiratory tract (URT) and then in the lower respiratory tract (LRT), and corresponding ex vivo/in vitro models aimed at investigating and comparing new strains of this virus are being developing now [1,2]. Mathematical modelling is a powerful tool for understanding complex biological phenomena [3,4], and we use a mathematical approach here to study infection propagation in space.

The SARS-CoV-2 virus enters the host from air mainly through cells of the URT—olfactory and respiratory nasal epithelial cells—using the ACE2 (angiotensin-converting enzyme 2) receptor and TMPRSS2 (transmembrane serine protease 2) [5,6]. ACE2 expression decreases from URT towards LRT [7], thus passage of infection to lungs takes some time. However, most of the damage this virus causes is in the lungs, where severe COVID-19 results in diffusive alveolar damage and multiple thrombosis leading to respiratory failure [8]. Other tissues (nervous, cardiovascular, etc.) are also susceptible to this virus [5]. Thus, current experimental studies of SARS-CoV-2 replication involve multiple cell lines (in addition, from different species), but cells from URT and LRT are of the greatest concern [1,2]. In particular, comparison of different strains infecting these lines may produce insights concerning their relative virulence, severity, predictions of epidemic progression, etc.

**Figure 2:**
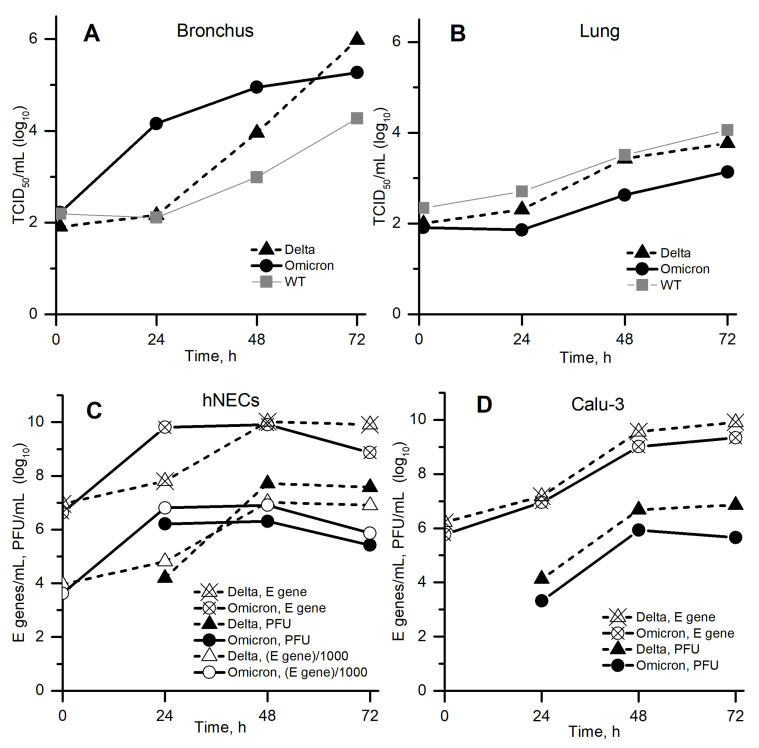
Viral replication kinetics: (**A**,**B**) in bronchus and lung cells [1]; (**C**,**D**) in nasal epithelial and human lung cells [2] (data were extracted from Figure 1 in preprint of [1] and from Figure 1 in preprint of [2]). (**C**) Open markers show E gene concentration (crossed markers) divided by 1000 to approximately match the corresponding PFU concentration.

The very recent SARS-CoV-2 variants of concern are Delta and Omicron [9]. Figure 2 shows the kinetics of replication of wild-type, Delta and Omicron variants in the URT and LRT cells reported by Hui et al. [1], Peacock et al. [2]. In both studies, a very interesting phenomenon was observed: Omicron got ahead of Delta in cells located above in the respiratory tract (A, C) during the first 1–2 days, but then, on the 2nd–3rd days, Delta overtook Omicron. In contrast, in cells located below in the respiratory tract (B, D), Delta got ahead during the whole time interval 1–3 days. While the second case seems to be obvious, the first one is not, and we aimed to study it closely. In particular, we are interested in the following questions regarding Figure 2A,C:

- Can these experiments be described by the mathematical model (the simplest possible one)?
- Could the situations of one strain overtaking another strain after some prolonged time interval be observed qualitatively if the spatial effects are taken into account?
- Could spatial effects be very important: for example, could they result in any new information that cannot be obtained in the homogeneous system?

In spatially distributed systems with highly non-linear kinetics, one can expect completely new phenomena principally impossible in homogeneous conditions: stationary patterns and travelling fronts/waves of concentration distribution. Classical examples include animal coat patterns, epidemic progression across populations, nerve action potential propagation, Belousov–Zhabotinskii reaction, calcium waves in cells, etc. [4]. A decade ago, autowave properties were found in blood coagulation in no-stirring conditions [10] after their first prediction in 1994 [11] and subsequent extensive experimental and theoretical research which used mathematical models of different complexity, from two to dozens of variables (reviewed in [12]). Recently, autowave spatial progression of viral infection in tissues was studied theoretically by Bocharov et al. [13] using rather general mathematical models of two equations (for virus and immune cell concentrations). Mathematical models of infection propagation in tissues are based on the same approaches as models of epidemic progression across populations, models of chemical and biochemical concentration waves, etc.: these are reaction–diffusion models, but frequently with time delay because virus release from an infected cell occurs some finite time after cell infection (in the case of modelling the immune response with cell proliferation, corresponding terms frequently contain time delays, too). On the one hand, using time delays simplifies model equations, but on the other hand delays are the greatest drawbacks of such models as they hamper both analytical and numerical studies.

Recent studies of autowave spatial infection spreading in cell cultures were also based on delay reaction–diffusion equations [14]. There, the conditions for infection propagation in terms of viral replication number and the formulas for the propagation speed were derived involving time delay as a parameter. In the present study, we use a similar approach, but (i) replace time delay with the intermediate cell sub-population—“exposed cells”—that is, infected but still not shedding the virus, and (ii) explicitly account for the decrease of free virus concentration concerning cell infection (which was neglected in previous studies). First, we obtain the new formulation for the viral replication number (which determines the condition for infection progression in space), estimates for the speed of spatial propagation of infection, and formulas for the final concentration of intact cells and the total (spatially-integrated) viral load. Second, we calibrate the model to recent ex vivo experimental data concerning replication of SARS-CoV-2 variants Delta and Omicron in cultures of human nasal epithelial cells reported by Peacock et al. [2]. Third, we numerically study spatial infection propagation in these cultures both in no-competition and in competition assumptions. We show that in the spatially distributed conditions, the Omicron variant, despite its much lower viral replication number, steady peak and total concentrations compared to those of Delta variant, can win the competition with Delta and completely suppress it.

## 2. Methods

### 2.1. Model Description and Governing Equations

Let us consider the scheme shown in Figure 1A. In the spatially distributed case, the system of equations corresponding to this scheme is:

(1a) $$\begin{array}{cc} \frac{dU}{dt} & =-aUV, \end{array}$$

(1b) $$\begin{array}{cc} \frac{dE}{dt} & =aUV-\gamma E, \end{array}$$

(1c) $$\begin{array}{cc} \frac{dI}{dt} & =\gamma E-\beta I, \end{array}$$

(1d) $$\begin{array}{cc} \frac{dV}{dt} & =-\rho \cdot aUV+N\cdot \beta I-\sigma V+D\nabla^{2}V. \end{array}$$

Here, variables *U*, *E*, and *I* stand for the concentrations of uninfected cells, “exposed” cells (infected but still not producing the virus), and infected cells (producing the virus), respectively, *V* stands for the concentration of virus particles in the medium, *D* is the diffusion coefficient of virus particles. *N* is the total number of virus particles produced by one cell during its life, N·β is the rate of virus production by one infected cell, ρ is the number of virions needed to infect one cell (usually only one virion is needed to infect a cell, but we keep ρ instead of 1 for generality and to study its influence on the results). Note that *V* and *N* account only for active virus particles in the medium, i.e., those that are able to infect a cell and self-replicate using it, but the total number of virus particles (which can be measured, for example, by PCR) can be much higher [2,15], see Section 2.4 below. The characteristic times of processes we account for are: cell infection, $\tau_{a}=\frac{1}{aU_{0}}$; E→I transition, $\tau_{E}=\frac{1}{\gamma }$; cell death (and viral shedding), $\tau_{I}=\frac{1}{\beta }$; viral death, $\tau_{V}=\frac{1}{\sigma }$. Boundary conditions for Equation (1d) are $V_{x}^{\prime }\left(\pm \infty \right)=0$. Initial conditions for Equation (1) are

$$\begin{array}{c} U\left(x,0\right)=U_{0},\;E\left(x,0\right)=0,\;I\left(x,0\right)=0,\;V\left(x,0\right)=\left\{\begin{array}{cc} V_{0}, & \mathrm{if}\;x\in \Omega_{0},\\ 0, & \mathrm{otherwise}, \end{array}\right. \end{array}$$

where $\Omega_{0}$ is some small but finite region onto which the virus has been inoculated (in calculations, we smooth the initial V(x,0) distribution in $\Omega_{0}$, see Section 2.5). When considering the homogeneous case of Equation (1), we omit the diffusion term and the *x* coordinate, passing to the corresponding ODE system with initial conditions $\left(U_{0},0,0,V_{0}\right)$.

### 2.2. Steady Travelling Solution

We are particularly interested in the steady travelling wave solution for Equation (1), which is established after some transition time period. Introducing the moving frame

ξ=x−ct,τ=t,

where *c* is the steady (i.e., established) wave speed, we denote u(ξ)=U(x,t), p(ξ)=E(x,t), q(ξ)=I(x,t), v(ξ)=V(x,t), where *u*, *p*, *q*, and *v* are independent on the new time τ. In new coordinates, Equation (1) become

(2a) $$\begin{array}{cc} -cu^{\prime } & =-auv, \end{array}$$

(2b) $$\begin{array}{cc} -cp^{\prime } & =auv-\gamma p, \end{array}$$

(2c) $$\begin{array}{cc} -cq^{\prime } & =\gamma p-\beta q, \end{array}$$

(2d) $$\begin{array}{cc} -cv^{\prime } & =-\rho \cdot auv+N\cdot \beta q-\sigma v+Dv^{\prime \prime }, \end{array}$$

where prime denotes the derivative with respect to ξ. Conditions at infinity are

$$\begin{array}{cc} u(+\infty ) & =u_{0}\equiv U_{0},\\ u(-\infty ) & =u_{f},\\ p(\pm \infty ) & =q\left(\pm \infty \right)=v\left(\pm \infty \right)=v^{\prime }\left(\pm \infty \right)=0. \end{array}$$

Below in this article, we derive the condition for the infection progression, estimates for the steady wave speed, *c*, and formulas for the relative concentration of uninfected cells after the front, $w=\frac{u_{f}}{u_{0}}$, and for the total virus concentration, $\tilde{v}=\int_{-\infty }^{+\infty }v\left(\xi \right)\;d\xi$, as functions of parameters.

### 2.3. Two-Strain Model with Competition for Cells

The scheme shown in Figure 1B describes the coexistence of two virus strains, $V_{1}$ and $V_{2}$, competing for the uninfected cells, *U*. The system of equations corresponding to this scheme is:

(3a) $$\begin{array}{cc} \frac{dU}{dt} & =-a_{1}UV_{1}-a_{2}UV_{2}, \end{array}$$

(3b) $$\begin{array}{cc} \frac{dE_{1}}{dt} & =a_{1}UV_{1}-\gamma_{1}E_{1}, \end{array}$$

(3c) $$\begin{array}{cc} \frac{dE_{2}}{dt} & =a_{2}UV_{2}-\gamma_{2}E_{2}, \end{array}$$

(3d) $$\begin{array}{cc} \frac{dI_{1}}{dt} & =\gamma_{1}E_{1}-\beta_{1}I_{1}, \end{array}$$

(3e) $$\begin{array}{cc} \frac{dI_{2}}{dt} & =\gamma_{2}E_{2}-\beta_{2}I_{2}, \end{array}$$

(3f) $$\begin{array}{cc} \frac{dV_{1}}{dt} & =-\rho_{1}\cdot a_{1}UV_{1}+N_{1}\cdot \beta_{1}I_{1}-\sigma_{1}V_{1}+D\nabla^{2}V_{1}, \end{array}$$

(3g) $$\begin{array}{cc} \frac{dV_{2}}{dt} & =-\rho_{2}\cdot a_{2}UV_{2}+N_{2}\cdot \beta_{2}I_{2}-\sigma_{2}V_{2}+D\nabla^{2}V_{2}. \end{array}$$

Here, indexes 1 and 2 correspond to strains 1 and 2 (Delta and Omicron in this article), respectively. Diffusion coefficients for two strains are supposed to be equal. Initial conditions for each strain are just like those in the one-strain model, with equal initial distributions.

### 2.4. Model Parameters and Comparison with Experimental Data

In this article, we use two sets of model parameters:

- Arbitrary non-dimensional values (typically, of the order of 1) for plotting the explanatory graphs when deriving analytical formulas in the Appendices. For this set, in numerical calculations we take $L=200,\;U_{0}=1$.
- Parameters which correspond to the SARS-CoV-2 variants Delta and Omicron replicating in human nasal epithelial cultures (hNECs) in the in vitro experiment [2] (see Table 1). We estimated them from physical reasons (e.g., *D*), derived/estimated from experimental results and kept fixed (e.g., $V_{0}$, $\tau_{a}$), or varied under the strict limitations as described below:
  1. The diffusion coefficient *D* was estimated using the Stockes–Einstein formula at 300 K assuming virus diameter 100 nm [16] and water viscosity.
  2. The experimental in vitro system in [2] is considered as homogeneous, because (a) only average concentrations are presented in that article, (b) the full size of the experimental system is 6.5 mm [17], which is comparable to the width of the front in our numerical calculations (about 1–3 mm, see Figure 5 below), and (c) substantial convection should be expected during inoculation and everyday sampling.
  3. Since the virus concentration determined by RT-qPCR for the E gene in [2] (see Figure 2C,D) was approximately 1000 times greater than the PFU concentration for all available time points and cell lineages (crossed vs. filled markers), that is only 1 out of 1000 viral particles was able to effectively infect a cell and reproduce with its help, we compared the model variable V(t) with the united data for $\frac{\mathrm{PFU}}{\mathrm{mL}}$ (filled markers) and $\frac{E\;\mathrm{genes}}{\mathrm{mL}}/1000$ (open markers). In particular, we estimated $V_{0}$ as $\frac{E\;\;\mathrm{genes}}{\mathrm{mL}}\left(t=0\right)/1000$.
  4. Initial cell concentration $U_{0}$ was limited in the range $\left[10^{4},10^{6}\right]\;\frac{\mathrm{cells}}{\mathrm{mL}}$.
  5. The characteristic time of infection ($\tau_{a}$) was assumed to be 1 h for both strains. Characteristic times if E→I transition ($1/\gamma_{1,2}$) and cell death ($1/\beta_{1,2}$) were limited as 2 h and 0.1 h from below, respectively. Virus death times ($1/\sigma_{1,2}$) were limited in the range [0.1, 100] h.
  6. To avoid large differences between the corresponding parameters of Delta and Omicron variants, the rations $\frac{\beta_{1}}{\beta_{2}}$, $\frac{\gamma_{1}}{\gamma_{2}}$, $\frac{\sigma_{1}}{\sigma_{2}}$ and $\frac{N_{1}}{N_{2}}$ were limited to the range of from $\frac{1}{10}$ to 10.
  7. Parameters $U_{0}$, $\gamma_{1,2}$, $\beta_{1,2}$, $\sigma_{1,2}$, and $N_{1,2}$ were determined by simultaneous best-fitting of two homogeneous versions of system (1)—for Delta and for Omicron variants—to the experimental data represented in Figure 2C from [2].
  8. After the fitting, all parameters were rounded up to 1 decimal digit, and those that were close to each other were set as equal.
  9. For the obtained set of kinetic parameters, in spatial numerical calculations (with diffusion) we set *L* = 2 cm or more to be able to track the transition of autowave to the steady propagation regime.

### 2.5. Numerical Methods

1. *Parameter estimation in the homogeneous case.* We used COPASI 4.34 [18] to estimate the model parameters $U_{0}$, $\gamma_{1,2}$, $\beta_{1,2}$, $\sigma_{1,2}$, and $N_{1,2}$ from the experimental data (Section 2.4). During these estimations, each initial value problem was solved using the LSODA method (relative tolerance $10^{-6}$, absolute tolerance $10^{-8}$). The parameter estimation task used the Differential Evolution algorithm (1000 generations, population size = 256) to minimize the sum of absolute differences of log(V) in the model and experiment at time points 0, 24, 48, and 72 h. Constraints for the parameter values and for their ratios described in Section 2.4 were applied. To fulfil the limitations on Delta/Omicron parameter ratios, two uncoupled versions of model (1)—for Delta and for Omicron strains—were solved simultaneously.

2. *Solution to the reaction–diffusion problems.* To integrate Equations (1) and (3), we used the previously developed package [10,19] based on the numerical methods described in [20]: Störmer–Encke’s method for space discretization (uniform mesh with 401 nodes, zero Neumann boundary conditions at x=0 and x=L) and the embedded Runge–Kutta–Fehlberg method of order 2(3) with automatic step size control for integration in time (mixed local error estimation with max norm, tol=0.001, fac=0.8, facmax=5). The activation zone was $\Omega_{0}=\left[0,\frac{L}{10}\right]$, and $V_{0}$ in this zone was multiplied by $2.257\cdot e^{-{\left(\frac{x}{L/20}\right)}^{2}}$ to reduce gradients at t=0, $x=\frac{L}{10}$ (thus, the average value of V(x,0) in $\Omega_{0}$ equaled $V_{0}$). The accuracy of the numerical method was controlled in two ways: (i) by solving the reduced Nagumo equation, $u_{t}^{\prime }=u^{2}\left(1-u\right)-hu+u_{xx}^{\prime \prime }$, followed by comparison of the steady speed with the theoretical value, $c_{theor}=\left[3\sqrt{(1-4h)-1}\right]/2\sqrt{2}$; relative discrepancy was <0.2% in the whole range of h∈[0,0.2] allowing autowave solution; (ii) by repeating some calculations of model (1) with ten times lower tolerance and two times lower spatial mesh size. For example, for Omicron parameters (Table 1) and ρ=1 relative changes of the steady speed and of the spatial-integrated virus concentration were 0.36% and 0.38%, respectively (relative to c≈0.0062 cm/h and $\tilde{v}\approx 0.93\cdot 10^{6}$ cells/cm${}^{2}$, see Table 2).

## 3. Results

### 3.1. Virus Replication Number Provides the Condition for the Infection Progression, $R_{v}>1$

At x→+∞, system (1) is in the steady state (ss) ${\left(U,E,I,V\right)}_{ss}=\left(U_{0},0,0,0\right)$. To study the stability of this steady state, we linearized equations around it (in the homogeneous case, neglecting the diffusion term). The stability is determined by the following Jacobian:

$$J_{ss}=\left|\begin{array}{ccc} -\gamma & 0 & aU_{0}\\ \gamma & -\beta & 0\\ 0 & N\cdot \beta & -\rho \cdot aU_{0}-\sigma \end{array}\right|=\left(R_{v}-1\right)\gamma \beta \sigma =\frac{R_{v}-1}{\tau_{E}\tau_{I}\tau_{V}}.$$

Here, we defined the virus replication number (VRN) as

(4) $$R_{v}=\frac{aU_{0}}{\sigma }\left(N-\rho \right)=\frac{\tau_{V}}{\tau_{a}}\left(N-\rho \right).$$

$R_{v}$ can be thought of as the ratio of virus production and death rates at the leading edge of the front, where $U\approx U_{0}$. Note that $R_{v}$ does not depend on the time of E→I transition, $\tau_{E}=1/\gamma$. The characteristic equation $|J_{ss}-\lambda E|=0$ reads as

$$\left|\begin{array}{ccc} -\gamma -\lambda & 0 & aU_{0}\\ \gamma & -\beta -\lambda & 0\\ 0 & N\cdot \beta & -\rho \cdot aU_{0}-\sigma -\lambda \end{array}\right|=0,$$

which gives

(5) $$\left(\lambda +\gamma \right)\left(\lambda +\beta \right)\left(\lambda +\sigma +\rho \cdot aU_{0}\right)=N\cdot aU_{0}\gamma \beta .$$

This equation for λ has one positive solution if $R_{v}>1$; otherwise, all its solutions are non-positive (see Appendix A.1). The existence of a positive λ means exponential growth of any perturbation of the steady state. Thus, the condition $R_{v}>1$ determines the instability of the steady state $\left(U_{0},0,0,0\right)$ of the homogeneous system, and infection progression in the spatially distributed system.

### 3.2. Estimates of the Steady Wave Speed

The speed of infection propagation in the steady travelling regime, *c*, is the eigenvalue to system (2). Analogous to the KPP problem, solutions exist for *c* in some range bounded from below, so the minimum possible speed should be found. After linearizing Equation (2) at ξ→+∞, where $u\to u_{0}$, we obtained the following estimate for the minimal wave speed (see Appendix A.2):

(6) $$c_{min}^{2}=\min_{\mu >\mu_{0}}F\left(\mu \right),\;\mathrm{where}\;F\left(\mu \right)=\frac{D\mu^{2}}{\mu +\sigma +\rho au_{0}-\frac{\gamma \beta }{\left(\mu +\gamma )(\mu +\beta \right)}Nau_{0}}.$$

The function F(μ) is negative at $0<\mu <\mu_{0}$ and positive at $\mu >\mu_{0}$, where $\mu_{0}$ is a discontinuity point, and the above equation corresponds to the minimum of the positive branch F(μ). We also obtained two explicit estimates of the minimal wave speed (see Appendix A.2):

(7a) $$\begin{array}{cc} c_{min}^{*2} & \approx 4D\frac{R_{v}-1}{\frac{1}{\sigma }+2\left(\frac{1}{\gamma }+\frac{1}{\beta }\right)\left(1+\frac{\rho au_{0}}{\sigma }\right)}, \end{array}$$

and, more precisely,

(7b) $$\begin{array}{cc} c_{min}^{**2} & \approx Dk_{12}\left[\sqrt{1+\frac{8}{k_{12}}\cdot \frac{R_{v}-1}{\frac{1}{\sigma }+2\left(\frac{1}{\gamma }+\frac{1}{\beta }\right)\left(1+\frac{\rho au_{0}}{\sigma }\right)}}-1\right], \end{array}$$

where

$$k_{12}=\frac{k_{1}}{k_{2}}=\frac{\gamma \beta +2\left(\gamma +\beta \right)\left(\sigma +\rho au_{0}\right)}{\gamma +\beta +2\sigma +2\rho au_{0}},\;\mathrm{and}\;\frac{\rho au_{0}}{\sigma }\equiv \frac{R_{v}}{\frac{N}{\rho }-1}.$$

**Figure A5:**
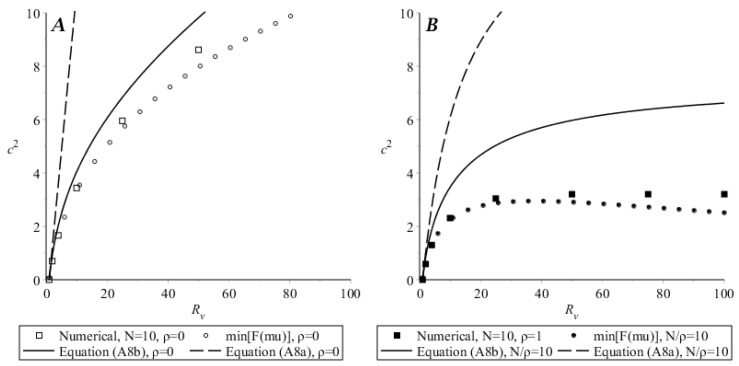
Steady autowave speed (squared) as a function of $R_{v}$ in the numerical solution (Equation (1), squares) and estimated using Equations (A5) (circles), (A8a) (dashed lines), and (A8b) (solid lines). (**A**) ρ=0, (**B**) ρ=1. Other parameters are the same as in Figure A2.

We compare these estimates with each other and with the numerical solutions in Appendix A.2 (Figure A5) for arbitrary values of parameters. After we calibrate the model to experimental data, we use Formulas (6) and (7b) for comparison with the numerical results for Delta and Omicron spatial propagation.

### 3.3. Equations for the Final Concentration of Intact Cells and the Total Spatial Viral Load

In spatially distributed conditions, concentrations of all components depend on spatial coordinates. In particular, in the autowave regime, virus concentration is zero before and after the autowave, and passes through the maximum in the wave region; the concentration of intact cells in the wave region (i.e., the region of nonzero virus concentration) drops from the initial to some final value (see Figure 5 below). Depending on aims and facilities, various concentration measures can be used, for example maximum and total virus concentration in the sample, final concentration of intact cells, and its ratio to the initial value. The relation between these values and the wave speed can be obtained by integration of Equation (2) over space (see Appendix A.3):

(8a) $$\begin{array}{cc} \ln w & =-R_{v}\cdot \left(1-w\right), \end{array}$$

(8b) $$\begin{array}{cc} \tilde{v} & =-\frac{c}{a}\ln w. \end{array}$$

**Figure 3:**
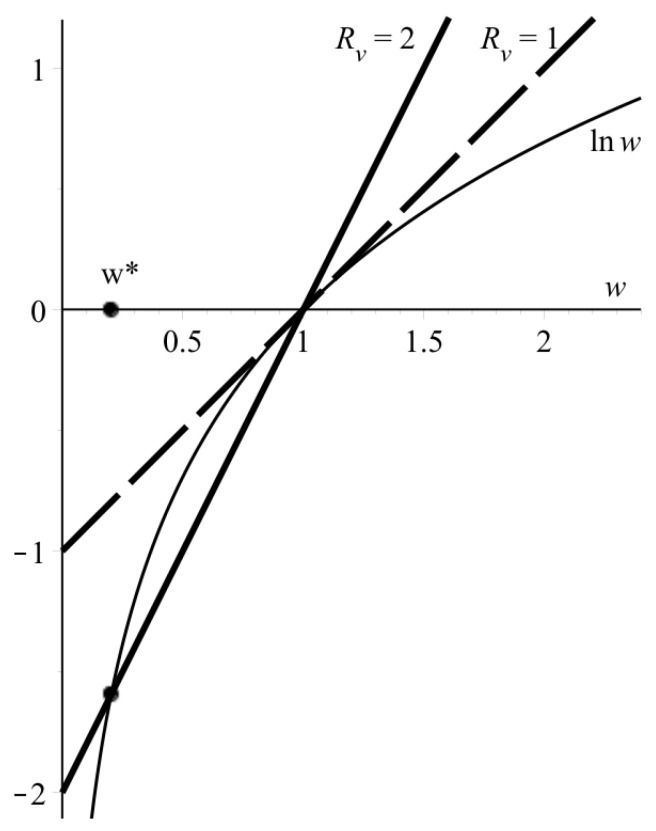
Graphical solution to Equation (8a) for two $R_{v}$ values. Thin line—lnw, bold lines—$R_{v}\cdot \left(w-1\right)$.

Here, $w=\frac{u_{f}}{u_{0}}\leq 1$ is ratio of the final to the initial concentration of intact cells, $\tilde{v}$ is total (spatially-integrated) virus concentration. If $R_{v}>1$, the transcendental Equation (8a) has two roots: 1 and $w^{*}$, where $0<w^{*}<1$ (Figure 3). Having found numerically the second root, $w^{*}$, one can determine the total spatial viral load $\tilde{v}$ using Equation (8b) provided that the wave speed *c* is found experimentally or theoretically (say, from Equation (6)). At $R_{v}>>1$, the root $w^{*}\approx 0$, so Equation (8) simplify to

(9a) $$\begin{array}{cc} \ln w & \approx -R_{v}, \end{array}$$

(9b) $$\begin{array}{cc} \tilde{v} & \approx \frac{c}{a}R_{v}. \end{array}$$

According to Equations (8) and (9), three main characteristics of infection, $R_{v}$, *c*, and $\tilde{v}$, appear to be closely linked (we consider *a* fixed in this study to simplify the analysis). However, they express different properties; that is why stains of the same virus may differ in any/all of them in various combinations, leading to unexpected consequences. This is shown below based on the comparison with the experimental data.

### 3.4. Homogeneous Case without Competition: Omicron Is “Quick” and Wins the Start but Delta Can Overtake It after 1–2 Days’ Lag

**Figure 4:**
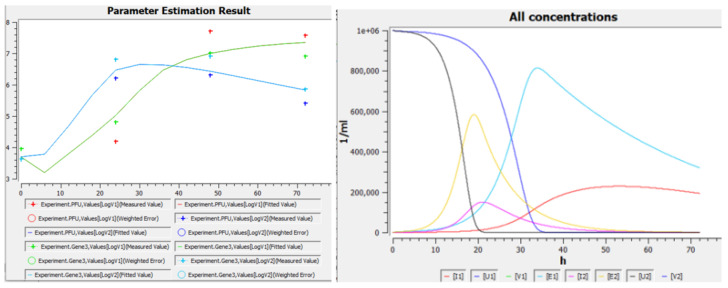
**Left**: Replication of the SARS-CoV-2 variants Delta (green + red) and Omicron (blue + cyan) in hNECs in the experiment [2] (markers, data are taken from Figure 2C as described in Section 2.4) and in the homogeneous version of the model (1) (lines). **Right**: concentrations of cells in the same solutions. Figures are screenshots from COPASI. Model parameters are listed in Table 1.

To study the case when Omicron wins the start but then Delta overtakes it (Figure 2A,C), we calibrated the homogeneous version of our model (Equation (1) with D=0) to one of these experiments (Figure 2C). Figure 4 (left) and Table 1 show results of this calibration, and Figure 4 (right) shows time dependencies of other variables (namely, concentrations of cells) in the same solutions. Obtained values of parameters suggest that Omicron is “weaker”: an infected cell produces five times less intact virions, and virion lifetime is six times smaller than for Delta. However, Omicron is “quicker”: its times of E→I transition and of virion release are five times smaller than for Delta. This quickness explains the fast start of Omicron compared to Delta in homogeneous conditions (Figure 2A,C and Figure 4). Later, after the transition period (1–2 days), Delta wins in homogeneous conditions (the same figures), apparently due to a greater *N* value. It is interesting to study the impact of spatial effects on this “number game”, because non-homogeneous conditions can be easily expected both in vivo (say, in the respiratory tract) and in vitro (say, in the plaque assay, or in other specially designed setups).

### 3.5. Spatially Distributed Case without Competition: Omicron Can Win the Race despite Low Concentration and $R_{v}$

**Figure 5:**
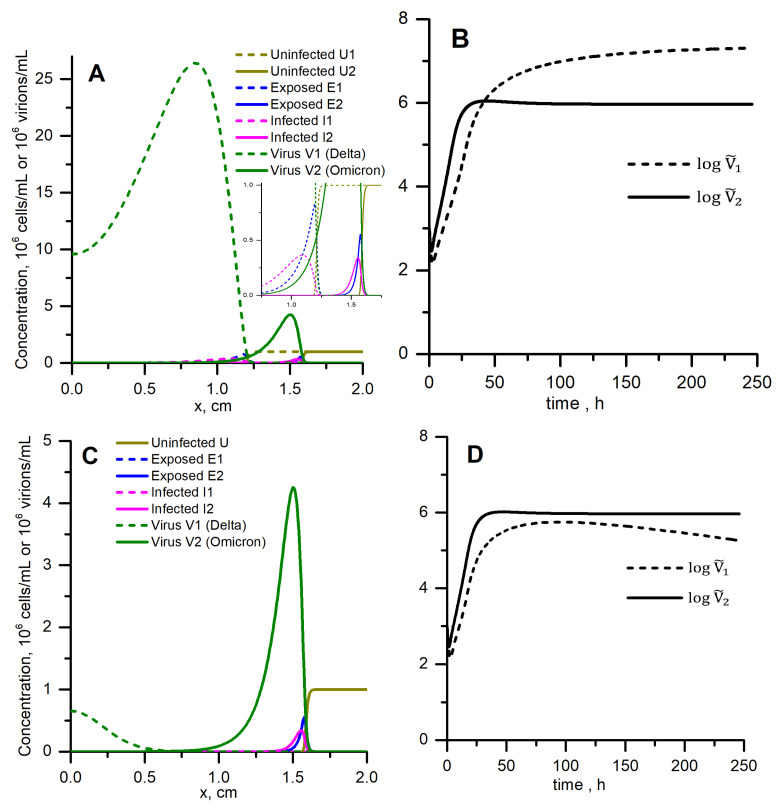
Spatial replication and propagation of Delta (dashed lines) and Omicron (solid lines) variants without competition for cells (**A**,**B**) and with competition (**C**,**D**). (**A**) Two uncoupled models given by Equation (1) were solved simultaneously. (**C**) Equation (3) were solved. (**A**,**C**) Solutions corresponding to the same distance (1.5 mm) travelled by Omicron autowave $V_{2}\left(x\right)$ are shown. (**B**,**D**) Logarithms of integrated virus concentrations vs. time are shown. ρ=1, all other parameters are listed in Table 1.

Solutions to the same equations with the same parameters but in the spatially distributed conditions are presented in Figure 5A. We show results for two strains on the common graph for comparison (but note that equations for two strains are uncoupled). The values of the steady autowave speeds and concentrations of virus are given in Table 2. Compared to Delta, Omicron has six times lower peak concentration and 26 times lower spatially integrated concentration. However, Omicron propagates 1.27 times faster: the steady speed is 0.0062 cm/h compared to 0.0049 cm/h for Delta. The kinetics of spatially integrated concentrations (Figure 5B) resemble homogeneous kinetics, where Omicron wins the start but then Delta overtakes it (Figure 4), but concentrations actually do not matter here: Omicron ultimately wins because it conquers more space, which means a greater number of cells or tissue volume infected. Note that the $R_{v}$ value for Omicron is also much lower than for Delta (Table 1).

**Figure 6:**
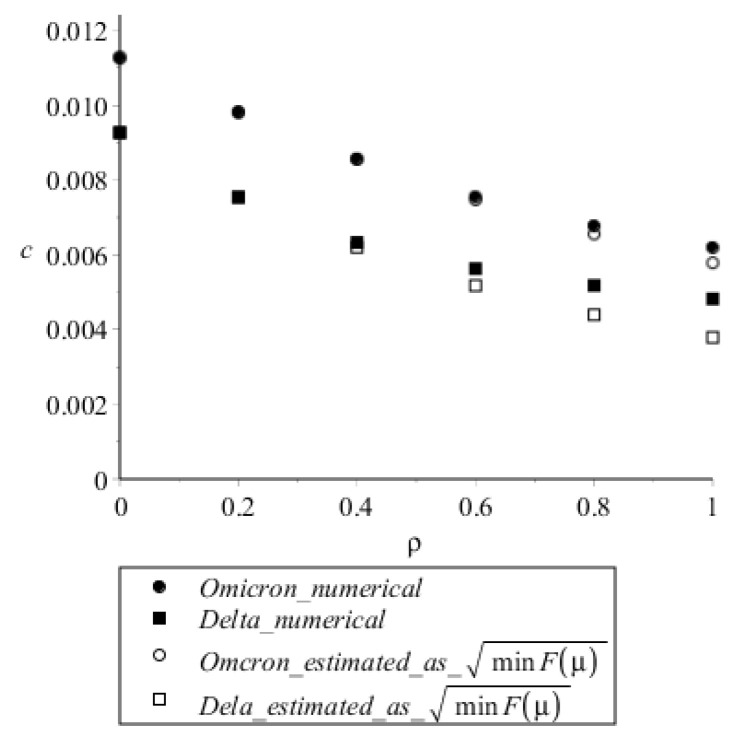
Steady autowave speed as a function of ρ, assuming that ρ can vary continuously (see Table 1 for other model parameters). Speed values for ρ=1 and ρ=0 are presented in Table 2.

Table 2 also allows one to compare the steady speeds with those estimated analytically from Equations (6) and (7b) for ρ= 1 and 0, and Figure 6 shows the gradual effect of ρ on speeds in numerical calculations and in Equation (6). For ρ=1, estimation by Equation (6) gives lower values than numerical calculations; however, at ρ=0, estimations and numerical calculations nearly coincide. We suggest that the discrepancy at ρ=1 is due to overestimation of the rate of virus concentration decrease upon cell infection in the linearized model which was used to derive the analytical estimation (see the Section 4).

### 3.6. Spatially Distributed Case with Competition: Omicron Can Win and Completely Suppress Delta

Solution to Equation (3) describing the competition of two virus strains is presented in Figure 5C. Equations for two strains are coupled now through the concentration of uninfected cells (the model suggests that the a cell cannot be co-infected by both strains). The calculation was stopped when the Omicron wave had travelled the same distance as in the no-competition situation (which is shown in Figure 5A). Compared to that case, the concentration of the Omicron strain is the same (solid line, note the difference in the y-scale), but the Delta strain is completely suppressed (dashed line). The steady speed of Omicron strain wave propagation is the same as in the no-competition situation, 0.0062 cm/h. The spatially integrated concentration of the Delta strain is lower than for the Omicron strain during the whole time period (Figure 5D).

## 4. Discussion

In this study, we use the simplest possible mathematical model of viral replication able to account for the delay of virus shedding (through introduction of the sub-population of “exposed” cells) and for the spatial non-uniformity of concentration distribution of all components. A typical solution in this system has autowave behaviour: being initially stimulated near one of the boundaries, the wave of virus concentration moves away from that site and finally goes into an autowave mode characterised by constant shape and velocity (Figure 5A). Spatial profiles of concentrations of exposed and infected cells also have the wave shapes, and the profile of uninfected cell concentration has a switch-off front shape (Figure 5A, insert). For values of parameters listed in Table 1, the width of the forward wave front is about 1–3 mm, so the characteristic distances on which the wave should be studied are centimeters (we set L≥2 cm). Experimental conditions (system size, convection, mixing, viscosity) and mode of virus transport between cells should alter the front width. For example, virus diffusion in a highly viscous medium or spreading through the cell-to-cell junctions should have a much lower effective diffusion coefficient, and thus the width of the wavefront should be smaller, leading to autowave behaviour detectable in smaller distances. In contrast, mixing or small size of the system should lead to spatial uniformity of concentrations, that is, to the homogeneous system lacking any spatial effects. That is why we use the homogeneous version of our model for calibration to experimental data obtained for hNECs in the MucilAir system (Figure 4, left).

We focus on viral replication in hNECs because these cells are important for SARS-CoV-2 entrance into the host as well as for the subsequent disease transmission, and because of intriguing kinetics reported in these cells for Delta and Omicron variants (Figure 2C). Experimental results show, and the homogeneous model is able to reproduce, that Omicron wins at the start (1st day), but in the next 1–2 days Delta overtakes it (Figure 4, left). After describing the homogeneous kinetics of Delta and Omicron replication in hNECs, we study the spatial propagation of infection in these cells in no-competition and in competition assumptions. Without competition, Omicron starts faster and its wave runs quicker, however with much lower maximal and total concentrations than Delta (Figure 5, top). When competition is allowed, Omicron ousts Delta and completely suppresses it (Figure 5, bottom). Thus, Omicron is “weaker but quicker”, it wins the competition with Delta using higher spatial speed.

These results are of great interest because qualitatively different properties of virus replication in cell culture or tissue can be distinguished: viral replication number $R_{v}$, viral concentration, either maximal $v_{max}$ or total (spatially integrated) $\tilde{v}$, and speed of spatial propagation of infection *c*. These properties appear to be closely linked in our simple model, see Equations (8) and (9). However, their “roles”, or interpretations, are qualitatively different. Consequently, correct understanding of these properties is necessary for designing appropriate experimental conditions and ways of processing experimental results.

From the analysis of Equation (5), we conclude that $R_{v}$ determines the very possibility of spatial propagation of infection. This conclusion seems unexpected because $R_{v}$ does not depend on either *D* or γ, see Equation (4). Mahiout et al. [14] obtained the same condition for autowave propagation, $R_{v}>1$, but their model included time-delay instead of “exposed” cells, and $R_{v}$ was also independent on the delay. This means that general results are robust concerning variations in particular model details. $R_{v}$ turned out to be not the parameter that determines the fate of spatial competition between strains. The same conclusion follows for the concentration of virus (both maximal and total): ability to obtain high concentration may not be important in competition (Figure 5). It is the speed of spatial propagation that determines the virulence and the fate of the competition between strains. The fastest strain conquers all the spatial area/volume of the culture or tissue.

Future studies should clarify whether this is a general conclusion for viral strain competition in tissues besides SARS-CoV-2 variants and hNECs. Another question which should be studied is the dependence of the competition fate on the initial conditions. As new strains appear due to mutations, from the very beginning they should compete with the old strain. Initial concentrations and spatial distributions should vary depending on location in the respiratory tract, person’s time from infection, etc. We suppose that the $R_{v}$ value may play a role in the survival of a new strain if this new strain is a small local patch surrounded by the old strain.

In this study, we were also faced with an interesting theoretical result: taking into account the relatively subtle process—the decrease of free virus concentration due to binding to the cell it infects, −ρ·aUV term in Equation (1d)—not only slightly decreases the speed of autowave propagation, but also decreases the estimated autowave speed relative to the one found numerically (Figure 6). The first effect is obvious, as any decrease of virus concentration should weaken the autowave. The second effect might not be so simple. It is not the numerical error, see Section 2.5. Thus, it could be some property of linearization of equations consisting in the substitution of u(ξ) with $u_{0}$, which is the basis for steady speed estimation (see Appendix A.2). Linearization increases the absolute value of the auv terms in the equations for concentrations of exposed cells and virus because $u_{0}>u\left(\xi \right)$. Moreover, u(ξ) rapidly drops in the wave front (Figure 5); thus, this replacement could have a great effect. However, linearization does not have any valuable effect on the speed at ρ=0, and the discrepancy increases with increasing ρ. Thus, the effect is likely due to the over-estimated rate of virus elimination in the linearized system. Indeed, from our parameters for Delta (Table 1), the rate constant for virus degradation is $\sigma_{1}=0.01$ h${}^{-1}$, but the quasi-1st order rate constant for virus binding, ρaU, at ρ=1 and $U=U_{0}$ is $aU_{0}=\frac{1}{\tau_{a}}=1$ h${}^{-1}$. Thus, at ρ>0, the linearization greatly increases the local virus elimination rate. The resulting effect on speed estimation is not great (Figure 6), but it should be noted that this effect is not attributed to numerical issues.

## 5. Conclusions

New experimental methods of viral investigation considering spatial effects, namely involving diffusion transport limitation and aimed at detecting the speeds and velocities of moving concentration waves, should be developed to obtain new insights concerning fundamentals of infection progression in tissues as well as between individuals. Practical application of these new methods will include facilitation of strain comparison and discovering new treatment options.

## Figures and Tables

**Table 1:** Model parameters for Delta and Omicron variants.

Parameter	Dimension	Delta	Omicron
		*Equal for both strains:*	
$V_{0}$	$\frac{\mathrm{virions}}{\mathrm{mL}}$	$5\times 10^{3}$	
$U_{0}$	$\frac{\mathrm{cell}}{\mathrm{mL}}$	$1\times 10^{6}$	
$\tau_{a}$	h	1	
$a\equiv \frac{1}{\tau_{a}\cdot U_{0}}$	$\frac{\mathrm{mL}}{\mathrm{cell}\cdot h}$	$1\times 10^{-6}$	
*D*	$\frac{{\mathrm{cm}}^{2}}{h}$	$1\times 10^{-4}$	
γ,β	$h^{-1}$	$\gamma_{1}=\beta_{1}=0.04$	$\gamma_{2}=\beta_{2}=0.2$
σ	$h^{-1}$	$\sigma_{1}=0.01$	$\sigma_{2}=0.06$
*N*	$1\equiv \frac{\mathrm{virions}}{\mathrm{cell}}$	$N_{1}=50$	$N_{2}=10$
$R_{v}=\frac{N-1}{\tau_{a}\sigma }$	1	$R_{v,1}=4900$	$R_{v,2}=150$

**Table 2:** Autowave speed $c\;(\frac{\mathrm{cm}}{h})$, spatially integrated viral concentration $\tilde{v}\;\left(10^{6}\frac{\mathrm{cells}}{{\mathrm{cm}}^{2}}\right)$ and maximal viral concentration $v_{max}\;\left(10^{6}\frac{\mathrm{cells}}{\mathrm{mL}}\right)$ for Delta and Omicron steady spatial autowave propagation.

	Delta	Omicron	Delta	Omicron
	ρ=1		ρ=0	
Numerical *c*	0.0049	0.0062	0.0094	0.011
$c_{min}$ as $\sqrt{min[F(\mu )]}$, Equation (6)	0.0038	0.0058	0.0093	0.011
*c* from Equation (7b)	0.0069	0.0088	0.016	0.015
Numerical $\tilde{v}$	24	0.93	46	1.88
$\tilde{v}=\frac{\sqrt{min[F(\mu )]}}{a}R_{v}$, Equations (6) and (9b)	19	0.87	45	1.65
Numerical $v_{max}$	26	4.3	27	4.9

**Figure A1:**
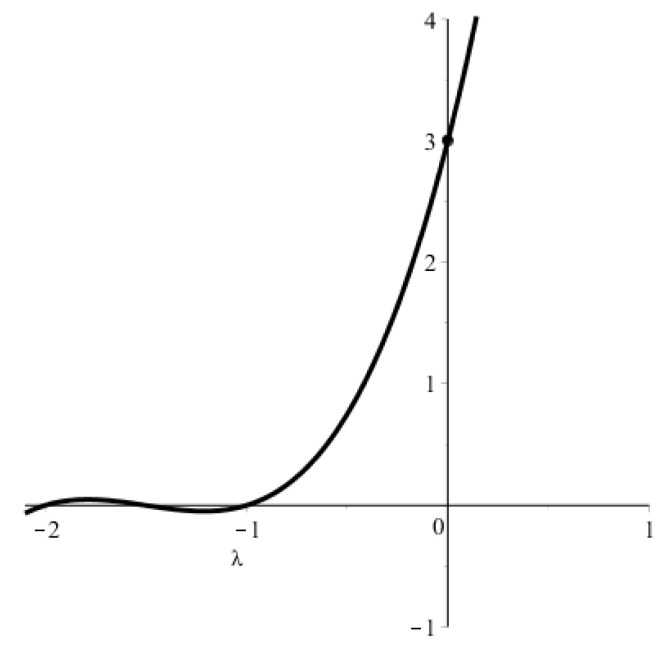
LHS of Equation (5) for γ=1.5, β=2, and $\sigma +\rho aU_{0}=1$.

**Figure A2:**
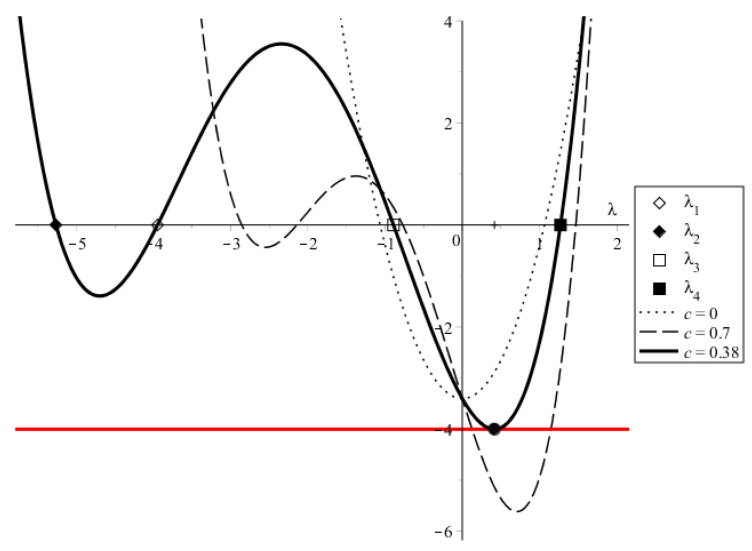
Solution of Equations (A2) and (A3). Black lines—LHSs, red line—RHSs. γ=1.5, β=2, σ=1, D = 1, $R_{v}=1.2$, $\frac{N}{\rho }=10$, and *c* values are specified.

**Figure A3:**
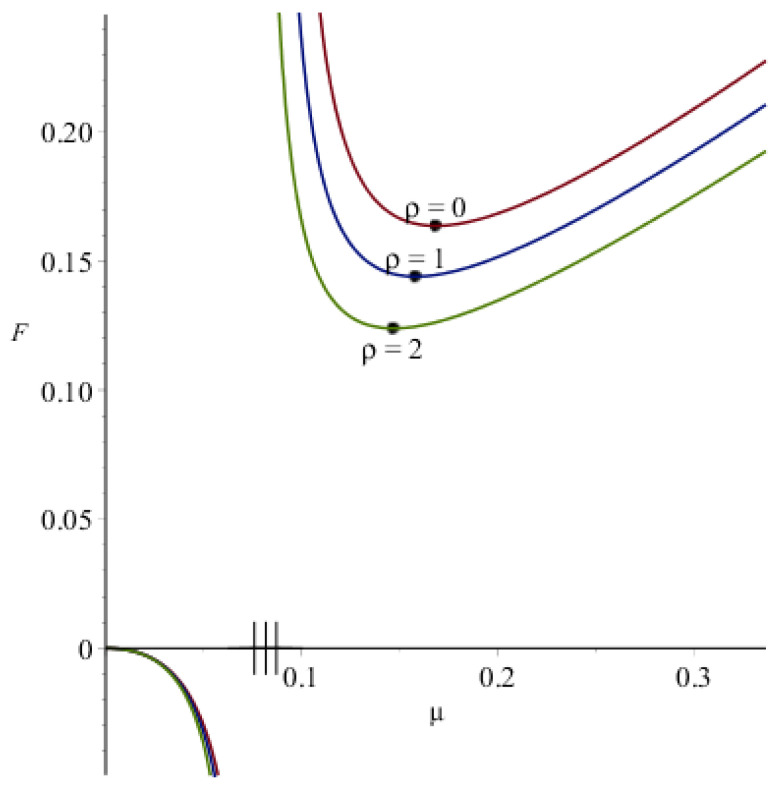
Function F(μ) from Equations (A4) and (6) at various ρ values. N=10, other parameters are the same as in Figure A2. Notches on the μ-axis denote discontinuity points $\mu =\mu_{0}$, closed circles denote minimum of the positive branch.

**Figure A4:**
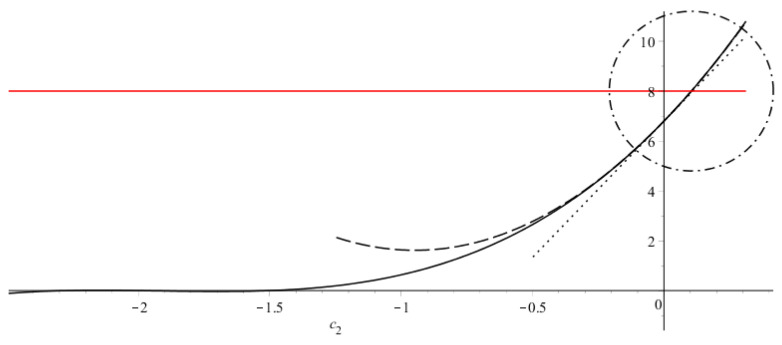
Solution to Equation (A7) (solid lines) and two its approximations in the $c_{2}\geq 0$ region giving $c_{2}^{*}$ (dotted line) and $c_{2}^{**}$ (dashed line). Parameters are the same as in Figure A2. Actual differences between solutions are hardly distinguished at these parameter values, however at larger $R_{v}$ all differences increase (see Figure A5).

## Data Availability

Not applicable.

## Abbreviations

The following abbreviations are used in this manuscript:

URT	upper respiratory tract
LRT	lower respiratory tract
hNECs	culture of human nasal epithelial cells
KPP or KPPF equation	Kolmogorov–Petrovskii–Piskunov–Fisher equation

## Appendix A

### Appendix A.1. Condition for Existence of a Positive Solution to Equation (5)

The condition $R_{v}>1$ for the existence of a positive solution to Equation (5) can be obtained using Figure A1, where the LHS (left-hand side) of this equation is plotted. The graph intersects the y-axis at $y=\gamma \beta (\sigma +\rho aU_{0})$. Thus, a positive solution exists if $\gamma \beta \left(\sigma +\rho aU_{0}\right)<NaU_{0}\gamma \beta \Leftrightarrow \sigma <\left(N-\rho \right)aU_{0}\Leftrightarrow R_{v}=\frac{aU_{0}}{\sigma }\left(N-\rho \right)>1$.

### Appendix A.2. Estimates of the Steady Wave Speed

Our estimations of $c_{min}$ are based on linearization of Equation (2) at ξ→+∞, where $u\approx u_{0}$. Substituting u(ξ) with $u_{0}$, we have:

(A1) $$\begin{array}{cc} -cp^{\prime } & =au_{0}v-\gamma p=\frac{v}{\tau_{a}}-\frac{p}{\tau_{E}},\\ -cq^{\prime } & =\gamma p-\beta q=\frac{p}{\tau_{E}}-\frac{q}{\tau_{I}},\\ -cv^{\prime } & =-\rho \cdot au_{0}v+N\cdot \beta q-\sigma v+Dv^{\prime \prime }=-\rho \frac{v}{\tau_{a}}+N\frac{q}{\tau_{I}}-\frac{v}{\tau_{v}}+Dv^{\prime \prime }. \end{array}$$

The right-hand sides (RHSs) of these equations are written first using the kinetic constants and then using the characteristic times. The solution of this system is close to the solution of system (2) in the leading front of the autowave. Let us look for the solution in the form $p=p_{1}e^{-\lambda \xi }$, $q=q_{1}e^{-\lambda \xi }$, $v=v_{1}e^{-\lambda \xi }$, where λ>0. Substituting these conjectures into Equation (A1), we obtain:

$$\begin{array}{cc} c\lambda p & =au_{0}v-\gamma p=\frac{v}{\tau_{a}}-\frac{p}{\tau_{E}},\\ c\lambda q & =\gamma p-\beta q=\frac{p}{\tau_{E}}-\frac{q}{\tau_{I}},\\ c\lambda v & =-\rho \cdot au_{0}v+N\cdot \beta q-\sigma v+D\lambda^{2}v=-\rho \frac{v}{\tau_{a}}+N\frac{q}{\tau_{I}}-\frac{v}{\tau_{v}}+D\lambda^{2}v. \end{array}$$

Eliminating *p*, *q* and *v*, we obtain the 4th degree equation for λ:

(A2) $$\begin{array}{cc} \left(c\lambda +\gamma \right)\left(c\lambda +\beta \right)\left[D\lambda^{2}-c\lambda -\sigma -\rho \cdot au_{0}\right] & =-N\cdot au_{0}\gamma \beta . \end{array}$$

If c>0, the LHS of this equation has four real roots (see Figure A2, black solid line and square markers):

$$\begin{array}{cc} \lambda_{1} & =-\frac{\gamma }{c}=-\frac{1}{c\tau_{E}},\\ \lambda_{2} & =-\frac{\beta }{c}=-\frac{1}{c\tau_{I}},\\ \lambda_{3,4} & =\frac{1}{2D}\left[c\mp \sqrt{c^{2}+4D\left(\sigma +\rho au_{0}\right)}\right]=\frac{1}{2D}\left[c\mp \sqrt{c^{2}+4D\left(\tau_{V}^{-1}+\rho \tau_{a}^{-1}\right)}\right]. \end{array}$$

At zero wave speed (c=0), Equation (A2) reduces to the equation

(A3) $$\begin{array}{cc} \gamma \beta \cdot [D\lambda^{2}-\sigma -\rho \cdot au_{0}] & =-N\cdot au_{0}\gamma \beta \end{array}$$

with roots of the LHS $\lambda_{3,4}^{0}=\mp \sqrt{\frac{\sigma +\rho \cdot au_{0}}{D}}=\mp \sqrt{\frac{\tau_{V}^{-1}+\rho \tau_{a}^{-1}}{D}}$ (Figure A2, dotted line). Equation (A3) has solutions $\lambda_{3,4}^{00}=\mp \sqrt{\frac{\sigma }{D}\left(1-R_{v}\right)}$, which requires $R_{v}\leq 1$ for $\lambda_{3,4}^{00}$ to be real. Thus, for $R_{v}>1$, Equation (A3) does not have real solutions (dotted line does not intersect the red one).

At λ=0, the LHSs of Equations (A2) and (A3) equal $-\gamma \beta (\sigma +\rho au_{0})$. For Equation (A3), this is the minimum LHS value, and it is greater than the RHS if $R_{v}>1$ (note that both the LHS and the RHS are negative). Consequently, for $R_{v}>1$ and small *c*, say $c\in [0,c_{min})$, Equation (A2) also does not have real solutions. Increasing *c* shifts the parabola peak right and down, and there exists a minimum value of the wave speed, $c=c_{min}$, for which Equation (A2) obtains a solution at $\lambda =\lambda_{0}>0$ (solid line, solid circle in Figure A2). For $c>c_{min}$ there are two solutions (dashed line). Thus, there exists a lower bound $c_{min}$ for possible wave speeds in solutions to the system (A1).

To find $c_{min}$, we use two approaches:

(1) Let us denote μ=cλ in Equation (A2):

$$\begin{array}{cc} \left(\mu +\gamma \right)\left(\mu +\beta \right)\left[D\frac{\mu^{2}}{c^{2}}-\mu -\sigma -\rho au_{0}\right] & =-N\cdot au_{0}\gamma \beta . \end{array}$$

Rearranging this equation, we obtain:

(A4) $$\begin{array}{c} c^{2}=\frac{D\mu^{2}}{\mu +\sigma +\rho au_{0}-\frac{\gamma \beta }{\left(\mu +\gamma )(\mu +\beta \right)}Nau_{0}}=\frac{D\mu^{2}}{\mu +\sigma \left(1+\frac{R_{v}}{\frac{N}{\rho }-1}\right)-\frac{\gamma \beta }{\left(\mu +\gamma )(\mu +\beta \right)}\sigma \frac{R_{v}}{1-\frac{\rho }{N}}}=F\left(\mu \right). \end{array}$$

The function F(μ) is plotted in Figure A3 (for several ρ values). This figure shows that there exists such $\mu_{0}>0$ that $F{\left(\mu \right)}_{|\mu >\mu_{0}}>0$. Moreover, the positive branch of the F(μ) graph has a single minimum. Consequently,

(A5) $$c_{min}^{2}=\min_{\mu >\mu_{0}}F\left(\mu \right),$$

which gives the exact estimate of the minimum wave speed in the system (A1).

(2) Let us note the approximate symmetry of the 4th order parabola near its peak, i.e., between $\lambda_{3}$ and $\lambda_{4}$ (Figure A2, black solid line in proximity to the red line). From the expression for $\lambda_{3,4}$, we conclude that

(A6) $$\lambda_{0}\approx \frac{\lambda_{3}+\lambda_{4}}{2}=\frac{c_{min}}{2D}.$$

Substituting this $\lambda_{0}$ into Equation (A2), we obtain

$$\begin{array}{cc} \left(\frac{c_{min}^{2}}{2D}+\gamma \right)\left(\frac{c_{min}^{2}}{2D}+\beta \right)\left(D\frac{c_{min}^{2}}{4D^{2}}-\frac{c_{min}^{2}}{2D}-\sigma -\rho \cdot au_{0}\right) & =-N\cdot au_{0}\gamma \beta . \end{array}$$

Denoting $c_{2}=\frac{c_{min}^{2}}{2D}>0$, we have

(A7) $$\begin{array}{cc} \left(c_{2}+\gamma \right)\left(c_{2}+\beta \right)\left(c_{2}+2\sigma +2\rho au_{0}\right) & =2N\cdot au_{0}\gamma \beta . \end{array}$$

Graphical solution to this equation is plotted in Figure A4 (solid lines). LHS (black solid line) has three negative roots, RHS = $y_{1}$ is constant (red line), and we look for their intersection at $c_{2}>0$ (circled region). At $c_{2}=0$, the LHS equals $y_{0}=2\gamma \beta \left(\sigma +\rho au_{0}\right)$. As $\frac{y_{0}}{y_{1}}=\frac{1-\frac{\rho }{N}}{R_{v}}+\frac{\rho }{N}$ and $\frac{\rho }{N}<1$, $y_{0}<y_{1}$ if $R_{v}>1$. Thus, a positive solution $c_{2}$ to cubic Equation (A7) exists if $R_{v}>1$. We can find the 1st and 2nd order estimates to this solution if we replace the positive branch (at $c_{2}\geq 0$) by the tangent line at $c_{2}=0$ or the parabola, respectively (black dotted and dashed lines):

$$\begin{array}{cc} y_{0}+k_{1}c_{2}^{*} & =y_{1},\\ y_{0}+k_{1}c_{2}^{**}+k_{2}c_{2}^{**2} & =y_{1}, \end{array}$$

where

$$\begin{array}{cc} k_{1} & ={\left[LHS\right]}_{|c_{2}=0}^{\prime }=\gamma \beta +2\left(\gamma +\beta \right)\left(\sigma +\rho au_{0}\right),\\ k_{2} & =\frac{1}{2}{\left[LHS\right]}_{|c_{2}=0}^{\prime \prime }=\gamma +\beta +2\sigma +2\rho au_{0}. \end{array}$$

Solving these linear and quadratic equations, we get two estimates for $c_{min}^{2}=2D\cdot c_{2}$:

(A8a) $$\begin{array}{cc} c_{min}^{*2} & =4D\frac{R_{v}-1}{\frac{1}{\sigma }+2\left(\frac{1}{\gamma }+\frac{1}{\beta }\right)\left(1+\frac{R_{v}}{\frac{N}{\rho }-1}\right)}, \end{array}$$

(A8b) $$\begin{array}{cc} c_{min}^{**2} & =Dk_{12}\left[\sqrt{1+\frac{8}{k_{12}}\cdot \frac{R_{v}-1}{\frac{1}{\sigma }+2\left(\frac{1}{\gamma }+\frac{1}{\beta }\right)\left(1+\frac{R_{v}}{\frac{N}{\rho }-1}\right)}}-1\right], \end{array}$$

where

$$k_{12}=\frac{k_{1}}{k_{2}}=\frac{\gamma \beta +2\left(\gamma +\beta \right)\sigma \left(1+\frac{R_{v}}{\frac{N}{\rho }-1}\right)}{\gamma +\beta +2\sigma (1+\frac{R_{v}}{\frac{N}{\rho }-1})}=\frac{1+2\frac{\tau_{I}+\tau_{E}}{\tau_{V}}\left(1+\frac{R_{v}}{\frac{N}{\rho }-1}\right)}{\tau_{I}+\tau_{E}+2\frac{\tau_{I}\tau_{E}}{\tau_{V}}\left(1+\frac{R_{v}}{\frac{N}{\rho }-1}\right)},$$

and

$$\frac{R_{v}}{\frac{N}{\rho }-1}\equiv \frac{\rho au_{0}}{\sigma }.$$

To compare these analytical results with each other and with numerical solution to the initial system (1), let us take values of parameters listed in the Figure A2 caption. In that figure, the solid line (corresponding to the minimal speed for which the autowave solution in the system (A1) exists) is plotted assuming c=0.38. This value was obtained from Equation (A5): minF(μ)≈0.144 (Figure A3, ρ=1 curve), so $c_{min}\approx \sqrt{0.144}\approx 0.38$. Estimates from Equations (A8) give $c_{min}^{*}\approx \sqrt{0.22}\approx 0.47$ and $c_{min}^{**}\approx \sqrt{0.21}\approx 0.46$. For the same parameters, numerical solution of Equation (1) has steady speed $c_{steady}=0.37$. Consequently, the estimate by Equation (A5) is quite precise, and both Equation (A8) over-estimate the speed. Here, the values of parameters corresponded to $R_{v}=1.2$ which is only a little above the boundary of autowave existence, $R_{v}=1$. Comparison in a wide range of $R_{v}$ values is given in Figure A5 for ρ=0 (A) and ρ=1 (B). Numerical solutions and estimates by Equation (A5) are close; however, discrepancy does exist and increases with increasing $R_{v}$. Estimates by Equation (A8b) are correct for $R_{v}$ from 1 to approximately 10, and estimates by Equation (A8a) are correct only for very small $R_{v}$ above 1. Thus, the estimate $\sqrt{min[F(\mu )]}$ gives the most precise speed value. Note that $R_{v}$ is actually the parameter of autowave existence (Section 3.1), and other parameters independently stay in formulas for F(μ), $c_{min}^{*}$, and $c_{min}^{**}$, so these ranges of $R_{v}$ values where estimates work well correspond to the given particular set of parameters.

### Appendix A.3. Derivation of Equations for the Final Concentration of Intact Cells and the Total Spatial Viral Load

Equation (8) for the final concentration of intact cells and the total spatial viral load can be derived as follows. From Equation (2a),

$$\begin{array}{cc} -c\frac{u^{\prime }}{u} & =-av,\\ c\int_{u_{0}}^{u_{f}}\frac{du}{u} & =a\int_{+\infty }^{-\infty }v\left(\xi \right)\;d\xi ,\\ c\ln\frac{u_{f}}{u_{0}} & =-a\int_{-\infty }^{+\infty }v\left(\xi \right)\;d\xi =-a\tilde{v}. \end{array}$$

From combined Equations (2a) and (2b),

$$\begin{array}{cc} -c{\left(u+p\right)}^{\prime } & =-\gamma p,\\ c\int_{u(-\infty )}^{u(+\infty )}du+c\int_{p(-\infty )}^{p(+\infty )}dp & =\gamma \int_{-\infty }^{+\infty }p\left(\xi \right)\;d\xi ,\\ c(u_{0}-u_{f})+0-0 & =\gamma \tilde{p}. \end{array}$$

From combined Equations (2a)–(2c),

$$\begin{array}{cc} -c{\left(u+p+q\right)}^{\prime } & =-\beta q,\\ c\int_{u(-\infty )}^{u(+\infty )}du+c\int_{p(-\infty )}^{p(+\infty )}dp+c\int_{q(-\infty )}^{q(+\infty )}dq & =\beta \int_{-\infty }^{+\infty }q\left(\xi \right)\;d\xi ,\\ c(u_{0}-u_{f})+0-0+0-0 & =\beta \tilde{q}. \end{array}$$

From Equation (2d),

$$\begin{array}{cc} -c\int_{v(-\infty )}^{v(+\infty )}dv & =-\rho a\int_{-\infty }^{+\infty }uv\;d\xi +N\beta \int_{-\infty }^{+\infty }q\left(\xi \right)\;d\xi -\sigma \int_{-\infty }^{+\infty }v\left(\xi \right)\;d\xi +D\int_{v_{\xi }^{\prime }\left(-\infty \right)}^{v_{\xi }^{\prime }\left(+\infty \right)}\;dv_{\xi }^{\prime },\\ -0+0 & =-\rho a\tilde{uv}+N\beta \tilde{q}-\sigma \tilde{v}+0-0. \end{array}$$

From combined Equations (2a) and (2d),

$$\begin{array}{cc} -c{\left(v-\rho u\right)}^{\prime } & =N\beta q-\sigma v+Dv^{\prime \prime },\\ -c\int_{v(-\infty )}^{v(+\infty )}dv+c\rho \int_{u(-\infty )}^{u(+\infty )}du & =N\beta \int_{-\infty }^{+\infty }q\left(\xi \right)\;d\xi -\sigma \int_{-\infty }^{+\infty }v\left(\xi \right)\;d\xi +D\int_{v_{\xi }^{\prime }\left(-\infty \right)}^{v_{\xi }^{\prime }\left(+\infty \right)}\;dv_{\xi }^{\prime },\\ -0+0+c\rho (u_{0}-u_{f}) & =N\beta \tilde{q}-\sigma \tilde{v}+0-0. \end{array}$$

Altogether, we have:

$$\begin{array}{cc} -c\ln\frac{u_{f}}{u_{0}} & =a\tilde{v},\\ c(u_{0}-u_{f}) & =\gamma \tilde{p}=\beta \tilde{q},\\ c\rho (u_{0}-u_{f}) & =N\beta \tilde{q}-\sigma \tilde{v}=\rho a\tilde{uv}. \end{array}$$

From the first equation, we obtain Equation (8b). Eliminating $\tilde{q}$ and $\tilde{v}$ and taking into account Equation (4), we obtain Equation (8a).
